# A Novel Thickness-Mode Broadband Piezoelectric Ultrasonic Transducer Design Based on Double-Layer Piezoelectric Structure and a Variable-Thickness Matching Layer

**DOI:** 10.3390/s26092610

**Published:** 2026-04-23

**Authors:** Qiao Wu, Aofeng Geng, Wenlin Feng, Meng Yao, Chao Hu

**Affiliations:** 1 School of Mechanical Engineering, Hubei University of Technology, Wuhan 430068, China; 102310045@hbut.edu.cn (A.G.); 102410153@hbut.edu.cn (W.F.); 102510048@hbut.edu.cn (M.Y.); 102510151@hbut.edu.cn (C.H.); 2 Key Lab of Modern Manufacture Quality Engineering, Hubei University of Technology, Wuhan 430068, China

**Keywords:** thickness-mode broadband ultrasonic transducer, non-uniform-thickness double-layer piezoelectric structure, variable-thickness matching layer

## Abstract

A novel broadband ultrasonic transducer design based on a non-uniform-thickness double-layer piezoelectric structure and a variable-thickness matching layer is proposed to overcome the limitations of conventional thickness-mode piezoelectric ultrasonic transducers, such as weak even-order harmonic responses and restricted bandwidth. The implementation of a non-uniform-thickness double-layer piezoelectric structure enables the simultaneous excitation and reception of ultrasonic signals containing both fundamental and second-harmonic frequencies. Furthermore, through the integration of variable-thickness matching layers with a backing material of non-uniform acoustic impedance, the dual resonant frequency responses are effectively merged into a broad bandwidth. The broadband transducer prototype is manufactured and characterized through electrical input impedance, time-domain pulse-echo signals, and corresponding frequency spectrum. Experimental results indicate a center frequency of 411.5 kHz, with dual resonant peaks observed near 298.6 kHz and 585.6 kHz, achieving a −6 dB relative bandwidth of 116%. The findings demonstrate that the self-developed broadband transducer is capable of effectively generating and receiving broadband signals containing both fundamental and second-harmonic components, thereby offering a new design strategy for broadband piezoelectric transducers.

## 1. Introduction

Broadband ultrasonic transducers have gained extensive application in fields including medical imaging [1,2,3], non-destructive testing [4,5,6], and underwater acoustics [7,8,9], owing to their advantages in pulse duration reduction, axial resolution improvement, and facilitation of subsequent signal processing and imaging reconstruction.

Current research on broadband ultrasonic piezoelectric transducers primarily focuses on piezoelectric material, multi-layer backing design, graded acoustic impedance matching layer design, broadband electrical impedance matching, and multi vibration mode structure. In terms of piezoelectric material improvements, the utilization of 1–3 PMN-PT piezoelectric composites or PVDF-based piezoelectric materials enables effective modulation of electromechanical coupling properties and equivalent losses, thereby enhancing frequency response characteristics [10,11]. For instance, Yang et al. successfully achieved a transducer with a −6 dB bandwidth of 135.9% by developing Sm-doped PMN-PT piezoelectric ceramics and fabricating 1–3 PMN-PT/epoxy composites, representing a 45.3% improvement over conventional PZT-5H materials [12]. Regarding polymeric piezoelectric materials, representatives such as P(VDF-TrFE) exhibit inherent broadband characteristics due to their low mechanical quality factors and acoustic impedance closer to that of propagation media. For example, the flexible large-area array transducer fabricated by Van Neer et al. based on PVDF-TrFE demonstrated a measured transmitting −6 dB bandwidth of approximately 78% around 8 MHz, while the receiving −6 dB bandwidth exceeded 107% [13].

Additionally, the backing layer exerts a direct influence on bandwidth through the regulation of equivalent damping and oscillation suppression. Although traditional tungsten/epoxy resin composite backing materials are well-established, they exhibit limited variation ranges in acoustic impedance and attenuation parameters, while their fabrication uniformity and repeatability are notably affected by processing variations [14,15]. Multi-layer backing solutions are proposed. For instance, Hou et al. developed a multi-layer backing structure composed of alternating epoxy resin and aluminum foil layers. By employing transmission line models and equivalent circuit analysis, the −6 dB bandwidth of a PMN-PT single-crystal transducer was increased from 27.72% to 33.44% [16].

However, it should be noted that the optimization of backing or piezoelectric materials is generally confined to the vicinity frequency of a single resonant peak and is constrained by the gain-bandwidth product, where bandwidth improvement often comes at the expense of transducer sensitivity. In conventional thickness-mode transducers, even-order harmonics are significantly suppressed, thereby limiting the potential for further bandwidth expansion. In addition, compared with PZT piezoelectric ceramics, PVDF-based materials exhibit superior receiving performance but do not possess notable advantages in terms of transmitting capability.

Acoustic impedance matching is another factor determining the forward energy transfer efficiency from the piezoelectric plates to the medium [17]. Gradient impedance matching layers have attracted considerable research attention. Bian et al. proposed a gradient matching layer with an exponential variation along the thickness direction, which was realized by filling 3D-printed tapered cavities with composite materials, achieving a transmitting voltage response bandwidth of 110% [18]. Hou et al. developed a gradient acoustic impedance material varying from 36.56 MRayls to 4.53 MRayls, and the fabricated transducer attained a −6 dB bandwidth of 71.24% [19]. Although gradient acoustic impedance matching layers demonstrate significant effectiveness in bandwidth improvement, the high requirements for material parameter controllability, geometric accuracy, and interfacial consistency lead to considerably high engineering implementation complexity.

Multi-resonant frequency or multi-mode coupling also represents an important approach to broadband ultrasonic transducer design. Lin et al. developed a multi-layer stepped piezoelectric structure, in which regions of varying thickness were radially partitioned to correspond to different resonant frequencies, achieving a broadband response with a center frequency of 10.54 MHz and a −6 dB bandwidth of 121% [20]. Zhang et al. developed a capsule-shaped multi-mode underwater acoustic transducer, attaining a −6 dB bandwidth of 90% and thereby significantly improving the bandwidth [21].

In addition to the structural design of the ultrasonic transducer itself, the introduction of an electrical impedance matching network between the excitation source and the ultrasonic transducer also constitutes an effective approach for enhancing the bandwidth of ultrasonic signals. To address broadband matching requirements, Yang et al. investigated various electrical matching network topologies based on the Smith chart method. The results indicate that electrical matching networks can significantly reduce the reflection coefficient and increase power gain and active power by approximately twofold [22]. For underwater piezoelectric transducers, Krishnakumar et al. proposed an LC-ladder broadband tuning network, which effectively improves power transmission characteristics over a wider frequency range compared to traditional narrowband tuning with shunt inductors [23]. Fu et al. introduced a broadband matching network design based on swarm intelligence optimization for low-frequency dipole acoustic logging transmitters. Experimental validation demonstrates that the operating bandwidth of the transducer can be expanded to approximately three times its original width after matching, along with a substantial increase in the excitation voltage amplitude [24].

In summary, current research on broadband ultrasonic transducers has achieved relevant progress in areas such as piezoelectric material, multi-layer backing, graded acoustic impedance matching layer, broadband electrical impedance matching, and multi-mode structure development. However, in the design of conventional thickness-mode piezoelectric transducers, the trade-off between bandwidth and sensitivity, as well as the suppression of second harmonics, remains unresolved. To address these issues, this paper proposes a novel design concerning a non-uniform-thickness double-layer piezoelectric structure with a variable-thickness matching layer: a double-layer piezoelectric structure is formed by stacking two piezoelectric plates with different resonant frequencies, enabling simultaneous excitation and reception of ultrasonic signals with dual resonant peaks containing both fundamental and second-harmonic components. Furthermore, by employing variable-thickness matching layers in conjunction with a non-uniform acoustic impedance backing, the aforementioned dual-resonant response is effectively merged into a broadband ultrasonic signal, thereby overcoming the bandwidth limitations of conventional thickness-mode piezoelectric transducers.

## 2. Fundamental Design and Simulation of Non-Uniform-Thickness Double-Layer Piezoelectric Structure

**Figure 1:**
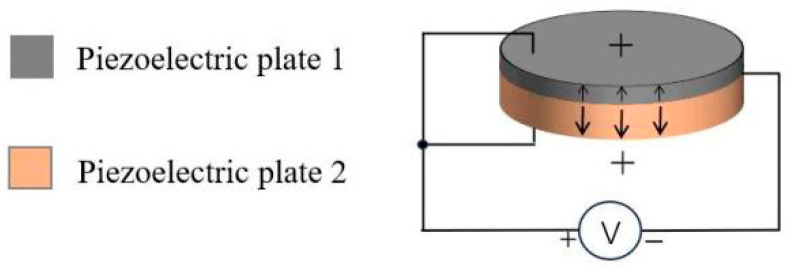
Non-uniform-thickness double-layer piezoelectric structure.

The non-uniform-thickness double-layer piezoelectric structure developed in this paper is achieved by bonding two piezoelectric plates of different thicknesses using epoxy resin, adopting a mechanical series and electrical parallel connection as illustrated in Figure 1. The arrows in Figure 1 indicate the polarization direction of the piezoelectric plate. The two piezoelectric plates are polarized along the thickness direction with opposite polarization orientations. The top and bottom surfaces are the positive electrodes, and the intermediate contact surface is the common negative electrode.

**Figure 2:**
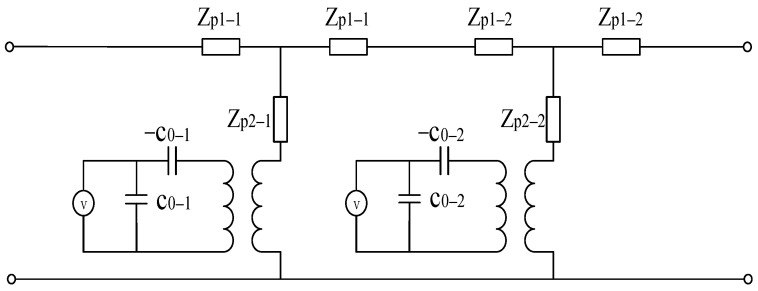
Equivalent circuit of double-layer piezoelectric structure.

The thickness mode vibration characteristics of the proposed double-layer structure under electrical excitation can be analyzed using an electromechanical equivalent circuit. Figure 2 presents the equivalent circuit model of the double-layer piezoelectric structure. Each piezoelectric plate is represented by a Mason equivalent circuit, with the mechanical ports of the two plates connected in series. When an electrical excitation signal is applied to the first piezoelectric layer, the second piezoelectric layer can be treated as an equivalent matching layer; conversely, when the excitation is applied to the second layer, the first layer can be treated as an equivalent backing layer. By applying identical electrical excitation signals simultaneously to both piezoelectric plates with different thicknesses, a dual-frequency ultrasonic signal containing both the fundamental and second harmonic components can be obtained.

**Figure 3:**
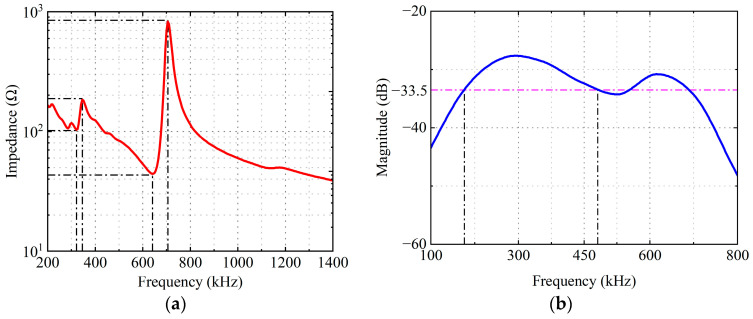
Simulation results of the double-layer piezoelectric structure: (**a**) the electrical input impedance; (**b**) the frequency spectrum.

A 2D axisymmetric finite element simulation model was established using COMSOL Multiphysics (version 6.3) to analyze the frequency response characteristics of the double-layer piezoelectric structure with non-uniform layer thickness. In the simulation model, PZT piezoelectric ceramic materials with center frequencies of 500 kHz and 1 MHz, respectively, were employed, both having a diameter of 30 mm. The selection of piezoelectric plates with center frequencies of 500 kHz and 1 MHz is based on their common usage as center frequencies for thickness-mode piezoelectric transducers in the field of non-destructive testing. In practice, as long as the thicknesses of the two piezoelectric plates are maintained in an approximately twofold relationship (with allowance for fine adjustment), the effects of the double-layer piezoelectric structure proposed in this work can be achieved. Figure 3a presents the simulated electrical input impedance curve, where two resonant and anti-resonant frequencies occur at 322 kHz, 345 kHz and 645 kHz, 705 kHz, respectively (As indicated by the black dashed line, the same applies below.).

Figure 3b shows the frequency spectrum of the double-layer piezoelectric structure obtained by simulation. The center frequency of the fundamental frequency is 291 kHz, and the center frequency of the second harmonic is 596 kHz, corresponding to the electrical input impedance curve. The fundamental resonant frequency corresponds to a half-wavelength equivalent thickness consistent with the total thickness of the two piezoelectric plates under thickness mode.

## 3. Finite Element Simulation of the Broadband Transducer Consisting of Variable-Thickness Matching Layers and Backing

Based on the non-uniform thickness double piezoelectric structure, a variable thickness matching layer was introduced to achieve acoustic impedance matching between the fundamental and second harmonics over a broad frequency band. The variable thickness matching layer is a thin circular plate with its thickness gradually varying along the radial direction. The outer edge has a thickness of 1.25 mm (corresponding to 1/4 wavelength of the resonant frequency of piezoelectric plate 1 in the double-layer piezoelectric structure; considering manufacturing capabilities, this is reduced to the 0.01 mm level), while the center has a thickness of 0.65 mm (corresponding to 1/4 wavelength of the resonant frequency of piezoelectric plate 2 in the double-layer piezoelectric structure). The variable thickness surface is designed as a sphere. The variable-thickness matching layer does not serve as an acoustic lens. Taking a focal length of 40 mm as an example, the thinnest part of a concave acoustic lens suitable for the proposed transducer would be 1.52 mm, while the thickest part would be 11.94 mm. Therefore, the thickness variation in the variable-thickness matching layer is far smaller than that of an acoustic concave lens. In fact, given the diameter of the variable-thickness matching layer of 30 mm, its thickness variation is very gradual, exerting a negligible influence on the radiated acoustic field of the transducer and essentially not functioning as a focusing lens. The matching layer material is a mixture of corundum and epoxy resin, with an acoustic impedance between that of piezoelectric materials and water. This reduces interface reflection and improves energy transfer, thereby enhancing transducer sensitivity. Considering that the thickness variation in the matching layer is relatively small compared to the transducer diameter, impedance gradient matching can be achieved over a broad frequency range without significantly disturbing the acoustic field distribution.

**Figure 4:**
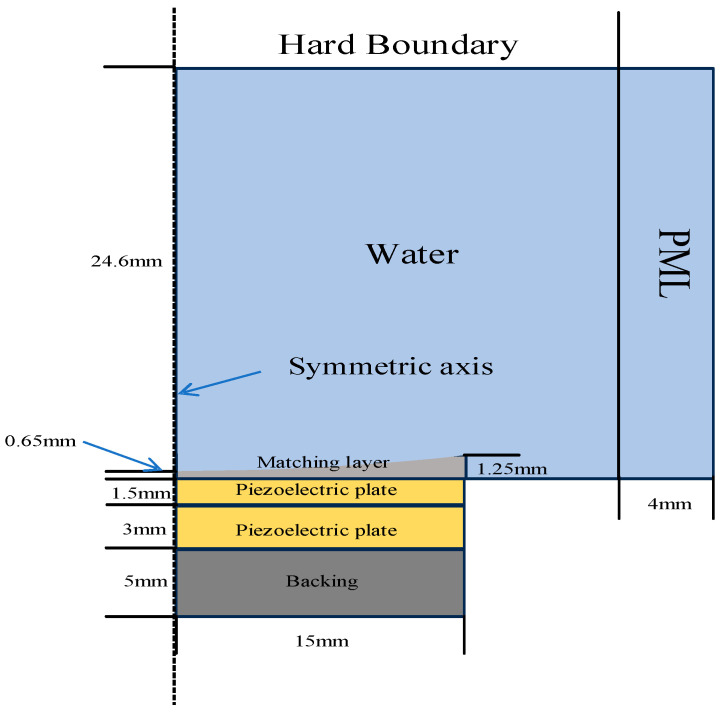
Schematic diagram of finite element simulation model of transducer.

Based on the above structural design, transient finite element simulation analysis was performed using COMSOL Multiphysics, and the simulation model is shown in Figure 4. The model is a 2D axisymmetric model, with the left side is the axis of symmetry. A variable-thickness matching layer is added to the front end of the double-layer piezoelectric structure, and a backing is added to the rear end. The medium is water, the right boundary is an absorbing boundary, and the top boundary is a reflecting boundary. To improve computational accuracy, the mesh size is set to 1/15 of the wavelength corresponding to a 1 MHz frequency. The time step is set to be less than the time required for the sound wave to pass through the smallest mesh element to ensure the stability and accuracy of the transient solution. A Gaussian pulse is used as the excitation source. The excitation signal is synchronously applied to both piezoelectric plates. The generated ultrasonic signal propagates forward in the water, is reflected after encountering a hard boundary, and is received by the transducer. The material coefficients used in the simulation are listed in Table 1.

**Figure 5:**
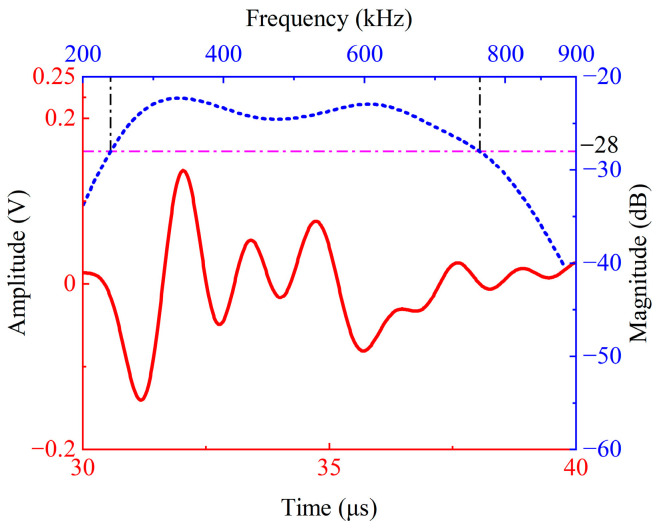
Simulation results of the broadband transducer with a variable-thickness matching layer and a backing layer.

Using the transducer’s terminal voltage as the received signal, the simulation results are shown in Figure 5, where the red line represents the time-domain received signal and the blue line represents the corresponding spectrum. For comparison, Figure 3b shows the simulation results of the double-layer piezoelectric structure. (The result in Figure 3b is obtained when the bottom edge of the double-layer piezoelectric structure is set as a low-reflection boundary, which replaces the function of the backing. Therefore, comparing Figure 3b and Figure 5 shows the role of the variable-thickness matching layer.) Whereas, Figure 5 shows the simulation results after adding a variable-thickness matching layer and a backing layer. Both sets of simulated time-domain waveform exhibit an echo wave at 29 µs, consistent with the reflection distance.

Spectral analysis of the simulated signals revealed that the spectrum contained both fundamental and second harmonic components, consistent with previous analysis. The center frequency of the double-layer piezoelectric structure was 291 kHz, with a −6 dB bandwidth of 306 kHz (the −6 dB relative bandwidth is 105%). The transducer with the variable-thickness matching layer had a center frequency of 430 kHz and a −6 dB bandwidth of 536 kHz (the −6 dB relative bandwidth is 125%). The introduction of the variable-thickness matching layer connected the two resonant peaks of the fundamental and second harmonics in the spectrum, increasing the transducer sensitivity by 5.5 dB while also improving the −6 dB bandwidth by 230 kHz (improving the −6 dB relative bandwidth by 20%). The simulation results validated the effectiveness of the broadband transducer design presented in this paper, providing a basis for subsequent transducer fabrication and experimental testing.

## 4. Fabrication and Experimental Testing of the Self-Developed Broadband Transducer

A broadband ultrasonic transducer is fabricated using a “double-layer piezoelectric structure + variable thickness matching layer” method. Based on simulation results, the double-layer piezoelectric structure uses two piezoelectric plates with center frequencies of 500 kHz and 1 MHz, respectively. The piezoelectric plates have a diameter of 30 mm, with thicknesses of 3 mm and 1.5 mm, respectively. The negative electrodes of the two piezoelectric plates are bonded together and led out as a common negative electrode, while the positive electrodes of the two piezoelectric plates are led out and connected together. The two piezoelectric elements are bonded together with epoxy resin.

**Figure 6:**
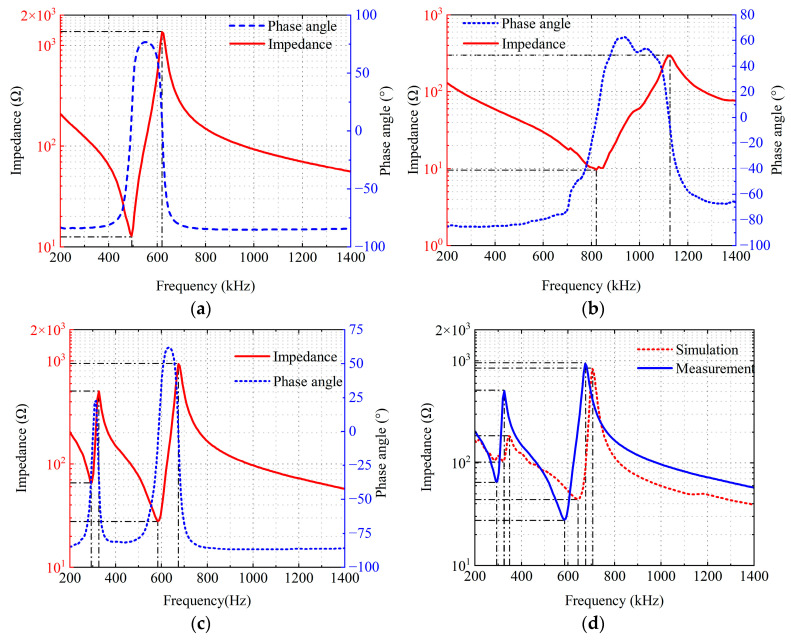
Electrical input impedance testing results: (**a**) 500 kHz piezoelectric plate; (**b**) 1 MHz piezoelectric plate; (**c**) double-layer piezoelectric structure; (**d**) simulation and experimental testing results of the double-layer piezoelectric structure.

The input impedance of the single-layer piezoelectric plates are measured using a KEYSIGHT E4990A impedance analyzer (Keysight Technologics, Santa Ros, CA, USA). After bonding, the input impedance of the double-layer piezoelectric structure is measured, and the results are shown in Figure 6. Figure 6a,b show the input impedance of the piezoelectric plates with center frequencies of 500 kHz and 1 MHz, respectively, while Figure 6c shows the input impedance of the double-layer piezoelectric structure. The solid red line represents the magnitude, and the dashed blue line represents the phase. Figure 6d compares the simulation and experimental results of the input impedance of the double-layer piezoelectric structure. It can be seen that the measured impedance curve of the double piezoelectric structure is mostly consistent with the simulation results in terms of the central frequency, number of resonance peaks and the overall trend. The main difference lies in the impedance magnitude. This magnitude deviation may stem from two aspects: firstly, simulation models are usually based on ideal boundaries and material parameters, and do not include non-ideal factors such as bonding layers, processing and assembly errors, and environmental disturbances; secondly, the equivalent acoustic parameters of different batches of piezoelectric composite materials vary to some extent. These deviations do not interfere with the main characteristics. Figure 6d presents the experimental electrical input impedance curve (the blue line), where two resonant and anti-resonant frequencies occur at 294 kHz, 325 kHz and 585 kHz, 675 kHz, respectively, showing less than 10% frequency deviation comparing to those of the simulation results (the red dashed line).

**Figure 7:**
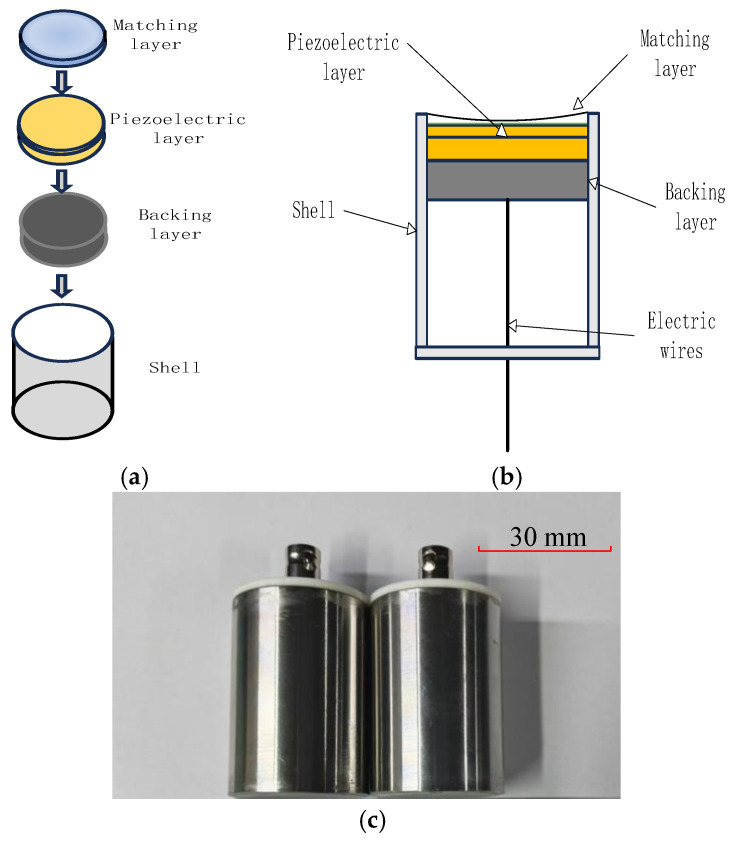
Self-developed broadband transducer: (**a**) the fabrication schematic; (**b**) cross-sectional structure; (**c**) the packaged transducer.

Figure 7 shows the fabrication schematic, cross-sectional structure, and the packaged transducer. A polytetrafluoroethylene (PTFE) spherical mold was used as the forming mold for the variable-thickness matching layer. The backing material is designed to accommodate a gradual variation in acoustic impedance along the thickness direction: the acoustic impedance is higher near the piezoelectric plate side and gradually decreases further away, which is achieved through a centrifugal sedimentation curing process. The matching layer material is a corundum/epoxy composite material with corundum particles of 400 mesh and a mass ratio of 2:1. The backing layer material is a tungsten/epoxy composite material, with tungsten powder particles of 325 mesh and a mass ratio (tungsten powder:epoxy) of 3.2:1. The piezoelectric material used is the 1–3 type piezoelectric composite material produced by Baoding Xinwei Electronics Technology Co., Ltd. (Baoding, China). Compared to the PZT piezoelectric ceramic in the simulation, the piezoelectric phase of the 1–3 type piezoelectric composite material is radially unconnected, further reducing the impact of introducing the variable-thickness matching layer on the transducer’s radiated acoustic field.

**Figure 8:**
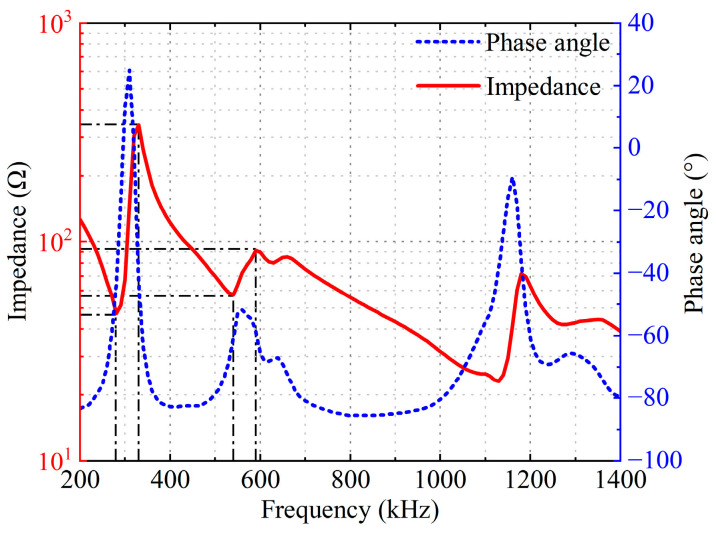
Experimental testing of the electrical input impedance of the self-developed broadband piezoelectric transducer.

The input impedance of the self-developed broadband transducer was tested using a KEYSIGHT E4990A impedance analyzer, as shown in Figure 8. As can be seen from Figure 8, the transducer exhibits the most significant impedance peak around 300 kHz (two resonant and anti-resonant frequencies occur at 295 kHz, 325 kHz and 585 kHz, 673 kHz, respectively.), with a noticeable change in the phase curve at this frequency, indicating the anti-resonance characteristics of the device’s main thickness mode. The magnitude of the impedance varies rapidly, forming a valley, while the phase transitions from capacitive to inductive, corresponding to the series resonance characteristics of the main mode.

Around 600 kHz, a second peak appears in the impedance curve, accompanied by a local change in the phase curve. This frequency band is approximately twice the size of the 300 kHz main peak and can be considered as the overall second-order thickness mode. The second-order thickness mode may couple with structural modes introduced by the matching layer, backing, or shell, resulting in a split in the impedance curve.

In addition, the phase curve in Figure 8 exhibits relatively prominent local fluctuations around approximately 1.2 MHz, while the impedance magnitude fluctuations are smaller. Such parasitic structural modes can be subsequently suppressed through optimization of the backing and matching layer.

Comparing the input impedance curves of the double-layer piezoelectric structure in Figure 6c, it can be seen that adding a variable-thickness matching layer and backing, despite introducing additional damping, does not significantly change the peak frequency of the transducer’s input impedance, maintaining similar frequency characteristics.

**Figure 9:**
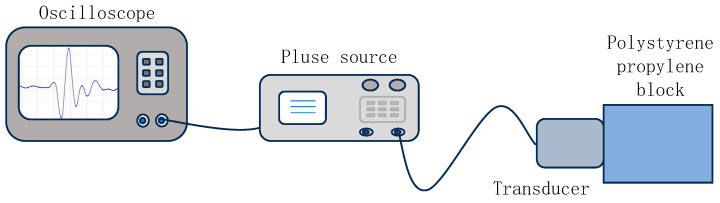
Schematic diagram of the experimental testing setup.

A pulse-echo experiment is conducted to test the time-domain received signal and spectrum of the self-developed broadband transducer. The pulse excitation signal is generated using a Shantou Ultrasonic GTS-8077PR (Shantou Ultrasonic Electronics Co., Ltd. Shantou, China) ultrasonic pulse excitation receiver. The schematic diagram of the experimental setup is shown in Figure 9. The pulse excitation settings are: pulse width 25 ns, voltage −100 V, gain 30 dB, excitation source internal resistance 50 W, and filtering set to 1 kHz–10 MHz. A Tektronix 3012B oscilloscope (Tektronix (China) Co., Ltd. Shanghai, China) is used to collect receiving signals. A 42 mm thick polystyrene (PSP) test block (Zhongshan Hanxuan Plastic Materials Co., Ltd. Zhongshan, China) is used for testing.

**Figure 10:**
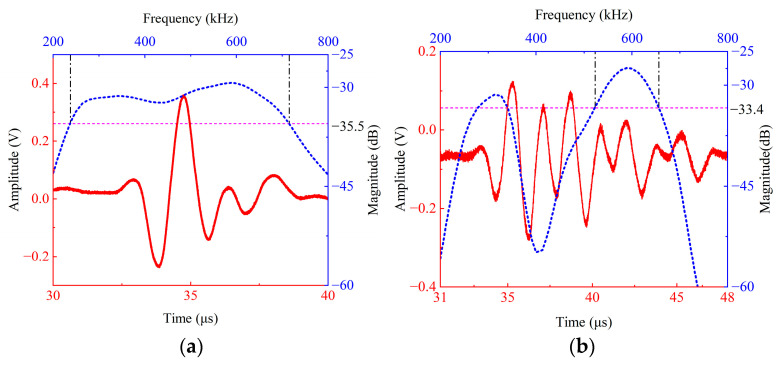
Experimental testing results of the time domain received signal (red line) and spectrum (blue line): (**a**) the self-developed broadband piezoelectric transducer; (**b**) the double-layer piezoelectric structure.

The test results are shown in Figure 10. Figure 10a shows the time-domain received signal and spectrum of the self-developed broadband transducer, while Figure 10b shows the time-domain received signal and spectrum of the double-layer piezoelectric structure under the same test conditions. The results show that the −6 dB relative bandwidth of the self-developed transducer is 116%, with two significant peaks appearing at 298.6 kHz and 585.6 kHz, corresponding to the fundamental response and its second harmonic component, respectively. This indicates that the structure can generate stable harmonic output under fundamental excitation conditions. Compared with the simulation results in Figure 6, the measured center frequency and the −6 dB relative bandwidth of the transducer are basically consistent.

Comparing the spectra of Figure 10a (complete transducer) and Figure 10b (dual piezoelectric structure only), the magnitude curve of the transducer is smoother after the introduction of the variable thickness matching layer and backing, the magnitude fluctuations near the main peak are suppressed, and the −6 dB relative bandwidth is significantly improved from 34% to 116%. According to the experimental results presented in Figure 10, the peak-to-peak values of the time-domain echo signals for the two configurations are 0.4 V and 0.6 V, respectively. It can be observed that, although the introduction of the backing brings certain losses, the signal sensitivity of the transducer is still improved to some extent due to the presence of the variable-thickness matching layer.

The experimental results show that the double-layer piezoelectric structure can achieve the generation and reception of ultrasonic signals with dual resonant peaks containing the fundamental wave and the second harmonic; after adding a variable thickness matching layer and backing, it can further achieve the generation and reception of broadband ultrasonic signals containing the fundamental wave and the second harmonic.

**Figure 11:**
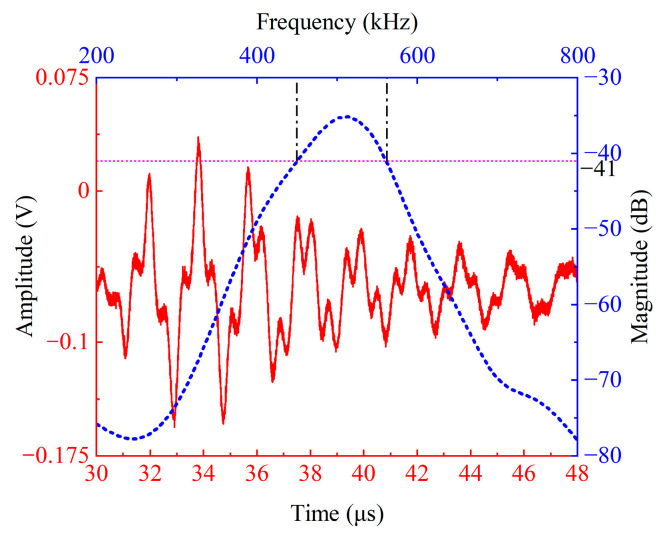
The comparative test results of commercial thickness mode transducers of the time domain received signal (red line) and spectrum (blue line).

A conventional thickness-mode ultrasonic transducer with the same diameter of 30 mm and a similar central frequency of 505 kHz manufactured by Changzhou Daobo Ultrasonic Electronics Co., Ltd. (Changzhou, China) is used for comparative testing under the same experimental conditions. The time-domain received signal (red line) and spectrum (blue line) are shown in Figure 11. Comparing Figure 11 and Figure 10a, the results show that the self-developed broadband transducer exhibits a shorter time-domain echo signal oscillation duration, faster waveform attenuation, a smaller detection blind zone, and higher axial resolution.

In terms of frequency domain characteristics, the spectrum of the commercial control probe mainly exhibits a single resonant peak with a center frequency of 505 kHz and a −6 dB relative bandwidth of 22%, displaying the narrowband response characteristics typical of a thick-mode transducer. In contrast, the self-developed broadband transducer has a −6 dB relative bandwidth of 116%, which is 94% higher than that of the commercial transducer. Its spectrum shows two resonant peaks corresponding to the fundamental and second harmonic components, reflecting characteristics distinct from other thick-mode broadband transducers. The results indicate that, under identical experimental conditions, the insertion loss improvement of the developed transducer and the conventional transducer is approximately 5.5 dB (given that the excitation pulse width is only 25 ns, it is approximately treated as an impulse function, and the insertion loss is derived from the frequency spectrum). It is thus evident that the proposed design does not substantially compromise transducer sensitivity. Experimental tests verify the effectiveness of the novel broadband ultrasonic transducer design method proposed in this paper.

## 5. Conclusions

This paper addresses the problems of weak even-order harmonic response and limited effective bandwidth in conventional thickness-mode piezoelectric transducers. A broadband ultrasonic transducer design approach combining a non-uniform-thickness double-layer piezoelectric structure with a variable-thickness matching layer is proposed and verified. The research includes structural design, finite element simulation, transducer fabrication, and performance testing. The main conclusions are as follows:

1. A non-uniform-thickness double-layer piezoelectric structure can achieve the generation and reception of ultrasonic signals with dual resonant peaks containing the fundamental and second harmonic frequencies. Experimental results show that using two piezoelectric plates with resonant frequencies of 500 kHz and 1 MHz, and fabricating a double-layer piezoelectric structure with electrical terminals connected in parallel and mechanical terminals connected in series, it is possible to achieve the generation and reception of ultrasonic signals with dual resonant peaks containing 298.6 kHz and 585.6 kHz, which is basically consistent with the simulation results.
2. Based on the double-layer piezoelectric structure, the introduction of a variable-thickness matching layer and backing improves the transducer’s magnitude-frequency response characteristics, further enabling the generation and reception of broadband ultrasonic signals containing the fundamental and second harmonic frequencies. Experimental results show that after introducing a backing layer and a spherical variable-thickness matching layer with a thickness gradually varying from 0.65 mm to 1.25 mm, the two resonant peaks with the fundamental and second harmonic frequencies as their center frequencies in the transducer’s magnitude spectrum are interconnected, increasing the transducer’s −6 dB frequency domain relative bandwidth to 116%, significantly improving the transducer’s bandwidth.
3. The broadband transducer developed in this paper has a center frequency of 411.5 kHz and a relative bandwidth of 116% in the −6 dB frequency domain. Compared with the conventional single resonant peak thickness mode commercial transducer, the results show that the −6 dB relative bandwidth of the self-developed broadband transducer is improved by 94%.

In summary, the design method of non-uniform-thickness double-layer piezoelectric structure + variable thickness matching layer proposed in this paper realizes the generation and reception of broadband ultrasonic signals containing fundamental and second harmonics without relying on complex multimodal or high-order structural designs. This provides a new approach for the design of thickness mode broadband ultrasonic transducers and has application prospects in fields such as harmonic imaging and nonlinear ultrasonic testing. Future work plans to employ it for ultrasonic detection of the relative nonlinearity coefficient in 316 stainless steel plates and pipes fabricated via the gas tungsten arc welding (GTAW) process. Furthermore, given that broadband transducers can be combined with techniques such as pulse compression to enhance the signal-to-noise ratio, subsequent research also intends to utilize the proposed transducer for the testing of highly attenuative materials.

## Figures and Tables

**Table 1:** Material coefficients of piezoelectric, matching and backing layers.

	Materials	Velocity (m/s)	Density (kg/m^3^)	Poisson Ratio
Matching layer	Corundum/epoxy resin	2500	1362	0.4
Piezoelectric layer	PZT-5H	4200	7500	0.31
Backing	Tungsten/epoxy resin	2000	3900	0.3

## Data Availability

The original contributions presented in this study are included in the article. Further inquiries can be directed to the corresponding author.
